# Genetic and Sonographic Insights into First-Trimester Fetal Cystic Hygroma: A Retrospective 30-Year Analysis Using 3D/4D Ultrasound and Cytogenetic Evaluation in Croatia (1993–2023)

**DOI:** 10.3390/genes16080980

**Published:** 2025-08-20

**Authors:** Petra Podobnik, Tomislav Meštrović, Mario Podobnik, Igor Lončar, Ivan Bertović-Žunec, Kristian Kurdija, Dženis Jelčić, Zlata Srebreniković, Slava Podobnik-Šarkanji

**Affiliations:** 1 Department of Obstetrics and Gynaecology, University Hospital Merkur, Zajčeva 19, 10000 Zagreb, Croatia; 2 Department of Obstetrics and Gynaecology, Podobnik Special Hospital, Ul. Sveti Duh 112, 10000 Zagreb, Croatia; 3 DA VINCI Polyclinic, Petrovaradinska Ulica 110, 10000 Zagreb, Croatia; 4 University Centre Varaždin, University North, 42000 Varaždin, Croatia

**Keywords:** cystic hygroma, first-trimester ultrasound, 3D/4D ultrasonography, prenatal diagnosis, cytogenetic analysis, chromosomal abnormalities, trisomy 21, genetic screening, fetal anomalies, spontaneous resolution

## Abstract

Background/Objectives: Cystic hygroma is a congenital lymphatic malformation often identified during early pregnancy and frequently associated with chromosomal abnormalities and adverse outcomes. We aimed to appraise the genetic and clinical characteristics of fetuses diagnosed with cystic hygroma in the first/early second trimester, assess the resolution patterns in chromosomally normal cases, and provide insights into prognosis—based on data collected over a 30-year period. Methods: A retrospective cohort study was conducted on 405 consecutive fetuses diagnosed with nuchal cystic hygroma between 8.0 and 14.0 weeks of gestation from 1993 to 2023 at two tertiary care centers. Diagnoses were established using high-resolution transabdominal and transvaginal 3D/4D ultrasonography. All cases underwent prenatal cytogenetic analysis, including karyotyping. Fetuses with a normal karyotype were observed through serial ultrasounds through the remainder of the pregnancy to verify the eventual resolution of hygromas. Both descriptive and inferential statistical methods were used, with *p* < 0.05 as a cut-off (two-tailed). Results: Of the 405 fetuses, 210 (51.9%) had chromosomal abnormalities, most commonly trisomy 21, while 195 (48.1%) had a normal karyotype. A significantly higher frequency of trisomy 21 was observed compared to other identified chromosomal abnormalities (*p* < 0.001). In the chromosomally normal group, 85 (43.6%) showed spontaneous resolution of the hygroma within four weeks, and these pregnancies resulted in phenotypically normal live births. Septated hygromas were significantly more frequent in the abnormal karyotype group (71.4%). Conclusions: The finding and diagnosis of cystic hygroma in first trimester and early second-trimester pregnancy represent a strong predictor of chromosomal aneuploidy and warrant comprehensive prenatal genetic testing and close follow-up. However, in the absence of genetic abnormalities and additional malformations, spontaneous resolution is common, and neonatal outcomes are generally favorable. Health systems should provide equitable access to genetic testing and fetal imaging to support accurate diagnosis and informed decisions.

## 1. Introduction

High-frequency ultrasonography, particularly when conducted transvaginally with 3D/4D imaging, significantly enhances the detection of fetal anomalies [1]. The introduction of transvaginal ultrasound has markedly increased the early identification of cystic hygroma in pregnancy (especially in the first and early second trimester) [2,3,4]. Cystic hygroma is often linked to both structural anomalies and chromosomal defects, although favorable neonatal outcomes have also been reported in some cases [5,6,7].

Fetal nuchal cystic hygroma is assumed to appear because of abnormal development of the lymphatic system [8]. Prenatal diagnosis during the first trimester has been well-documented, with an incidence of up to 1% [1,5,6]. While the diagnosis in the second trimester is also possible, such cases are more commonly associated with Turner syndrome and, less frequently, with other chromosomal abnormalities [9,10]. Spontaneous resolution has been observed in fetuses with normal karyotypes, as well as in those with trisomy 21, Turner syndrome, and Roberts syndrome [11,12,13].

Due to the potential severity and variable prognosis of cystic hygroma, its prenatal detection warrants comprehensive follow-up, including detailed anatomical surveys and genetic testing [14]. In cases where chromosomal abnormalities are identified, multidisciplinary counseling involving obstetricians, geneticists, and neonatologists is essential to offer prospective parents clear guidance regarding possible prognoses and outcomes, as well as management options [15]. Early diagnosis also allows for consideration of additional investigations, such as fetal echocardiography, to assess for associated congenital heart defects, which are commonly seen in affected fetuses [16].

The principal aim of this retrospective observational analysis was to evaluate the association between chromosomal abnormalities and cystic hygromas detected during the first or early second trimester of pregnancy, with a particular focus on the frequency and type of aneuploidies and pathogenic copy number variants. The study also aimed to examine the prognostic significance of ultrasound features such as septation and anatomical location of the hygroma, and to assess the potential for spontaneous resolution in euploid fetuses. An additional objective was to describe the clinical outcomes of affected pregnancies over a 30-year period at two tertiary care centers in the Republic of Croatia.

## 2. Materials and Methods

The investigation was conducted as a retrospective observational study over a 30-year period (1993–2023) at two tertiary care centers: the Department of Obstetrics and Gynaecology, University Hospital Merkur, and the Department of Obstetrics and Gynaecology of the Podobnik Special Hospital, both located in Zagreb, Croatia. Demographic, clinical, ultrasound, and cytogenetic data were extracted from electronic records and standardized reporting forms.

The study included participants in whom cystic hygroma was identified during the first or early second trimester of pregnancy using 3D/4D transvaginal ultrasound. The inclusion criteria encompassed all fetuses with a confirmed finding of cystic hygroma by transabdominal or transvaginal ultrasound (3D/4D ultrasound) within the defined gestational window in the first and early second pregnancy trimester. Exclusion criteria included cases with incomplete karyotyping, missing follow-up ultrasound data, or coexisting major anomalies without definitive imaging features of cystic hygroma.

Ultrasonographic examinations were conducted for obstetric purposes, including estimation of gestational age, evaluation of vaginal bleeding, or referral for fetal karyotyping. Referrals were often due to advanced maternal age (37 years or older at the time of delivery) or the detection of cystic hygroma by a primary care provider.

Initial ultrasound assessments were performed using Toshiba SAL 77-B equipment (Toshiba Medical Systems Corporation, Otawara, Japan), followed by Toshiba Aplio XG ((Toshiba Medical Systems Corporation, Otawara, Japan) at the University Hospital Merkur. In later years, Voluson 8 and Voluson 10 systems were used at the Department of Obstetrics and Gynaecology of the Podobnik Special Hospital. Transabdominal scans were conducted with 5 MHz or 3.75 MHz transducers, while transvaginal examinations employed 2–9 MHz multifrequency endovaginal probes with 3D/4D imaging capabilities. Of note, while the diagnosis of cystic hygroma can be reliably established using high-quality 2D ultrasound, 3D/4D imaging was employed to improve spatial visualization of the lesion, assess septations and facilitate differential diagnosis from other posterior neck anomalies. Also, 3D/4D reconstructions were utilized for enhanced documentation and counseling purposes.

For the purposes of this study, cystic hygroma was defined as a prominent, anechoic or hypoechoic, septated or non-septated cystic mass (≥3 mm), typically located in the posterior cervical (nuchal) or occipital region and separated from the skin surface by a distinct outer membrane. Unlike increased nuchal translucency, which is a physiological subcutaneous fluid collection confined to the nuchal region in the midsagittal plane (and is a space rather than a discrete mass), cystic hygroma often extends beyond the anatomical limits of nuchal translucency, may involve bilateral or lateral neck regions, and is considered a pathological finding resulting from abnormal lymphatic development. Septation was specifically evaluated and documented in each case. Diagnoses were based on real-time imaging and confirmed through static 3D reconstructions, and associated anomalies were assessed in all suspected cases. All ultrasound scans were reviewed by experienced fetal medicine specialists. For consistency, gestational age of the fetus was estimated based on crown-rump length (CRL) measurement.

Cytogenetic analysis was conducted in all cases via transabdominal chorionic villus sampling. In pregnancies that were electively terminated, autopsies were performed. A normal karyotype was defined as a chromosomal complement consistent with the standard 46,XX or 46,XY configuration, without detectable structural or numerical abnormalities. An abnormal karyotype referred to any deviation from the normal chromosomal pattern, including aneuploidies, structural rearrangements or pathogenic copy number variants. In addition to conventional karyotyping, chromosomal microarray and targeted gene panels (including Noonan syndrome panels) were offered in selected cases. Advanced testing was performed when cystic hygroma was associated with one or more additional structural malformations, when family history suggested a potential hereditary syndrome, or when conventional karyotyping was normal but sonographic features raised suspicion for an underlying genetic disorder.

In fetuses with small, non-septated hygromas and a normal karyotype, weekly ultrasonographic follow-ups were conducted until resolution. Resolution was defined as complete disappearance of the cystic lesion on two consecutive ultrasounds. In these cases, resolution typically occurred within 4 to 6 weeks after diagnosis, with the newborns presenting as phenotypically normal at delivery. Serial ultrasound evaluations—including fetal echocardiography and screening for other malformations—were conducted at 12, 16, 20–24, and 30–34 weeks of gestation.

Descriptive statistics were used to summarize categorical and continuous variables. To assess whether the distribution of abnormal versus normal karyotypes differed significantly across chromosomal categories, a chi-square test of independence was performed. For the purposes of this analysis, five diagnostic groups were considered: trisomy 21, trisomy 18, trisomy 13, Turner syndrome, and a composite “other” group that included all remaining chromosomal abnormalities. A *p*-value of less than 0.05 was considered statistically significant (two-tailed). Statistical analysis was performed in R, version 4.3.3 (R Foundation for Statistical Computing, Vienna, Austria).

The study was approved by the Ethics Committees of University Hospital Merkur (No. 0.5.10.1997. N 133) and the Department of Obstetrics and Gynaecology of the Podobnik Special Hospital (No. 06.03.2008. N 13). Written informed consent was obtained from all participants undergoing invasive prenatal procedures.

## 3. Results

Over the three-decade period from 1993 to 2023, a total of 405 pregnancies were identified in which the fetus was diagnosed with cystic hygroma during the first trimester or the early part of the second trimester of gestation. Of these, 200 cases were managed at the Department of Obstetrics and Gynaecology, University Hospital Merkur, and 205 at the Podobnik Department of Obstetrics and Gynaecology, both in Zagreb, Croatia. The average gestational age at which the diagnosis was established was 11.5 weeks, with the observed range spanning from as early as 8.0 weeks to as late as 14.0 weeks of pregnancy.

In 305 of the pregnancies, the expectant mothers were referred for chorionic villus sampling as a result of either advanced maternal age or suspicious non-invasive prenatal testing results. In the remaining one hundred cases, the diagnosis was established following referral from primary care providers who had detected fetal cystic hygroma using transabdominal or transvaginal ultrasound.

Chromosomal analysis was performed in all 405 cases using chorionic villus sampling. An abnormal karyotype was identified in 210 fetuses (51.9%), while 195 (48.1%) had a normal karyotype. Among the abnormal cases, 154 were diagnosed before 13 weeks of gestation and 56 after 13 weeks. In women aged 37 years or older, 150 fetuses had an abnormal karyotype. In contrast, among younger women, 105 fetuses had a normal karyotype and 90 had abnormal results.

Within the group of fetuses exhibiting abnormal karyotypes, 80 were diagnosed with trisomy 21, 47 with trisomy 18, 33 with trisomy 13, and 30 with monosomy X (Turner syndrome). Three additional cases showed mosaicism with a 45,X/46,XY karyotype, confirmed via amniocentesis at 16 weeks of gestation. Five fetuses presented with pathogenic copy number variants. These included 10q26.12q26.3 deletion, 22q11.2 deletion and duplication, 5q21.1q34 deletion, and 46,XX with deletion at 2q13. Additionally, five fetuses were diagnosed with Noonan syndrome through a combination of conventional karyotyping, microarray analysis, as well as a targeted Noonan syndrome gene panel. A statistically significant difference was observed in the frequency of in the frequency of trisomy 21 compared to other mentioned abnormalities (chi-square = 64.82, df = 4, *p* < 0.001) (Table 1).

Regarding anatomical location, 204 of the cystic hygromas were positioned posteriorly in the nuchal region, 200 were lateral in the cervical region, and one case involved an anterior location. Septation was observed in 115 of 189 fetuses with abnormal karyotypes, corresponding to 60%. The remaining 21 fetuses with abnormal karyotypes had non-septated hygromas. Among the euploid fetuses, 15 had septated hygromas without any additional structural abnormalities. In contrast, 15 fetuses with septated hygromas and chromosomal abnormalities had associated malformations, including exomphalos, hydrocephalus with pleural effusion, generalized hydrops and cardiac anomalies in five cases. These pregnancies were electively terminated at 15 weeks of gestation.

**Figure 1:**
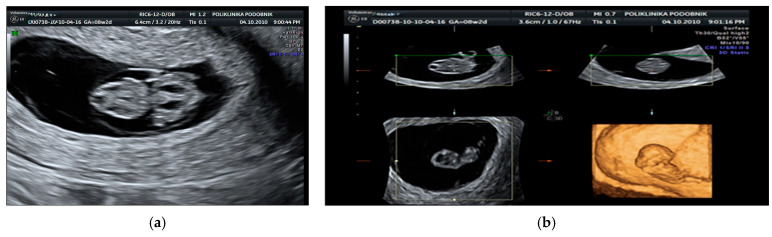
Longitudinal view of a non-septated cystic hygroma with associated hydrops fetalis and bilateral hydrothorax at 8.2 weeks of gestation. (**a**) Corresponding 3D transvaginal ultrasound image. (**b**) Fetal karyotyping revealed Turner syndrome. The pregnancy was electively terminated at the parents’ request.

**Figure 2:**
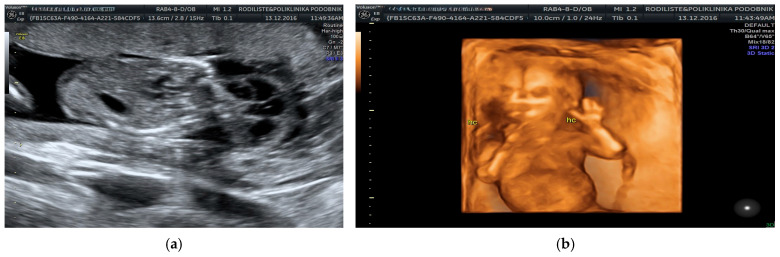
Transvaginal sonographic scan showing bilateral septated cystic hygroma in a fetus at 12.4 weeks of gestation. (**a**) Three-dimensional transvaginal ultrasound image of the same fetus. (**b**) Cytogenetic analysis revealed trisomy 21.

**Figure 3:**
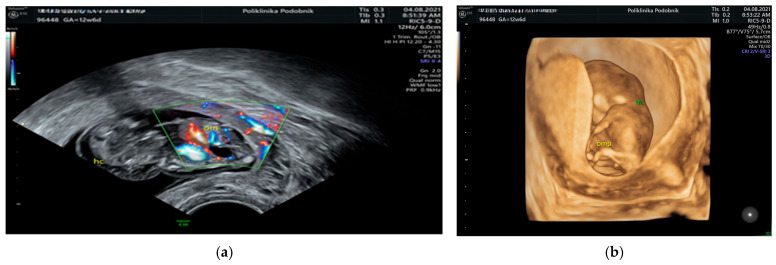
Ultrasonographic image of a septated fetal cystic hygroma with associated omphalocele using color Doppler imaging (**a**) and corresponding 3D transvaginal ultrasound. (**b**) The fetus was at 12.6 gestational weeks and karyotyping revealed trisomy 13.

All 210 pregnancies with confirmed chromosomal abnormalities were electively terminated, as were 142 pregnancies with normal karyotypes, based on parental request. Among the euploid fetuses, 58 cases (27.6%) showed spontaneous resolution of the hygroma within four to six weeks after diagnosis, with the newborns displaying no phenotypical abnormalities at delivery. In three additional euploid cases, the hygroma resolved initially, but severe oligohydramnios developed later. In two of these cases, polycystic kidneys and absent bladder filling were identified at 20 weeks, leading to pregnancy termination. Figure 1, Figure 2 and Figure 3 illustrate representative first-trimester cases of fetal cystic hygroma with associated anomalies—i.e., Turner syndrome, trisomy 21 and trisomy 13—captured through transvaginal and 3D ultrasound imaging.

## 4. Discussion

This research reaffirms the link between fetal nuchal cystic hygroma, when detected in the first or early second trimester of pregnancy, and the presence of chromosomal abnormalities. In addition, it demonstrates that transvaginal sonography in the first trimester can be effectively used to detect supplementary indicators (or markers) of chromosomal anomalies. Our findings align with earlier research showing that identifying fetal cystic hygroma in the first trimester is linked to a heightened likelihood of chromosomal abnormalities (Table 1). Notably, in our cohort of 405 pregnancies, chromosomal abnormalities were detected in 51.9% of cases with cystic hygroma, with trisomy 21 being the most frequent, followed by trisomies 18 and 13, and monosomy X. This substantial proportion underscores the clinical significance of early detection and reinforces the value of comprehensive prenatal genetic evaluation following sonographic diagnosis of cystic hygroma.

Cystic hygromas are congenital lymphatic system malformations characterized by fluid-filled cavities, most often located in the neck region. They usually stretch from the upper portion of the back of the skull downwards and inwards toward the prominent lateral neck muscle and comprise two symmetrical cavities separated by the midline nuchal ligament [17,18]. Around day 40 of embryonic development, paired jugular lymph sacs connect to the internal jugular veins, ultimately forming the terminal portions of the right lymphatic duct and the thoracic duct. According to the jugular lymphatic obstruction sequence theory, cystic hygroma develops when this connection does not form properly, resulting in lymph accumulation in neck tissues and sac enlargement [17,18]. Van der Putte confirmed this hypothesis almost half a decade ago [19], observing absent jugular venous connections in fetuses with cystic hygroma and generalized edema.

In cases of defective lymphatic vessel formation, the jugular lymphatic sacs become distended, leading to the buildup of lymph in the adjacent tissues. Cystic hygromas can diminish and resolve spontaneously if alternative lymphatic pathways form or if a connection to the jugular veins is eventually established [17,18,19]. Larger lesions are frequently separated by a central septum. This variant must be distinguished from other masses in the cranio-cervical area, such as meningomyelocele, encephalocele, teratoma, familial nuchal blebs, and haemangiomas [20,21,22,23,24,25]. In cases where a minute cystic hygroma is detected in the nuchal area, the possibility of a pseudomembrane should also be considered—most notably when the suspected abnormality is not confirmed on follow-up sonographic examinations [22,23,25].

First-trimester hygromas differ from those observed later in pregnancy, as the degree of cystic alteration is generally less advanced, septations are observed less often, while early signs are often limited to the appearance of a simple, elevated membrane akin to a localized thickening that is clearly set apart from the back portion of the fetal neck [26]. Careful differentiation is required to distinguish these findings from ultrasonographic artifacts or the amnion, which at this stage may still be structurally separate from the chorion until approximately 12 to 14 weeks of gestation.

In cases where the abnormality disappears, it is possible that the small cystic hygroma was reabsorbed or that the initial sonographic finding represented dorsal specular reflections from the skin surface during the first examination. Cystic formations in the fetal neck come in a wide range of sizes. Cullen et al. [27] described “nuchal blebs” as non-septated cystic lesions smaller than 3 mm, which typically resolve before the 11th week of gestation. A cystic elevation measuring 6 mm or greater from the skin surface to the membrane’s outer edge has been suggested as the minimal diagnostic threshold for cystic hygroma during the second trimester [13,20,27] (see Table 1 and Table 2). Although fetal head-to-trunk volume ratio was not assessed in our cohort, this parameter has been shown to correlate with major chromosomal abnormalities [28] and could be integrated into future prospective studies to enhance risk stratification and prenatal counseling.

As already highlighted, our findings indicate that 51.9% of fetuses displaying ultrasound evidence of cystic hygroma in the first or early second trimester possessed an abnormal chromosomal makeup, which is a clinically relevant finding (see Table 1). This aligns closely with the pooled data reported by Shulman et al. [29], who found a 46.9% incidence of chromosomal abnormalities in similar cases. Their study further supports the strong link between nuchal cystic hygromas detected in early gestation and a high incidence of aneuploidy. In our cohort, trisomy 21, 18, and 13 were more frequently observed in the first-trimester group. Fetuses with a 45,X karyotype (Turner syndrome) accounted for only 15.6% of the abnormal cases, which is consistent with the report by Cullen et al. [27], where only four instances of monosomy X were identified among fifteen cases of fetal aneuploidy linked to first-trimester cystic hygroma.

This observation is in contrast with cytogenetic findings in cystic hygromas diagnosed in the second or third trimester, where Turner syndrome is reported as the most prevalent chromosomal abnormality [6,19,20,25,30]. For example, Chevernak et al. [31] identified a 45,X karyotype in 11 of 15 fetuses with cystic hygroma identified during the gestational period of 18 to 29 weeks. Such cases of second-trimester cystic hygroma are commonly linked to an unfavorable prognosis.

In their review of 153 documented cases of fetal cystic hygroma, Abramowitz et al. [32] reported that, among the 110 cases with available karyotypes, 72.7% had chromosomal abnormalities. Notably, none of the fetuses with abnormal karyotypes survived, whereas five infants survived in the normal karyotype group—three of whom had significant medical complications related to their hygromas. Similarly, in a study by Cohen et al. involving 164 cases, 120 pregnancies (73.2%) were electively terminated at the request of the parents, while 37 fetuses (22.6%) experienced intrauterine death [33]. Among the 44 pregnancies that continued, only 3 (7%) resulted in live births, 2 of which involved neonates with normal karyotypes. In the subset of cases with cystic hygroma that resolved spontaneously in the second trimester, as reported by Chevernak et al. [31], no survivors were observed, further underscoring the poor prognosis associated with second-trimester presentations.

In our cohort, whether or not septations were present in the cystic hygroma showed no correlation with the fetal chromosomal profile. This finding is consistent with the results of Johnson et al. [6], Cullen et al. [27] and Shulman et al. [29], but contrasts with the conclusions drawn by Bernstein et al. [5] and Bronshtein et al. [34], who reported a significant association. Based on our findings, we do not consider the presence or absence of septations as a determining factor in guiding counseling or reassuring patients that the fetal karyotype is likely normal, nor do we consider it sufficient grounds to avoid additional prenatal diagnostic work-up. We assert that identifying any fetal cystic hygroma via ultrasound during the first trimester—especially if accompanied by other malformations—should be considered a marker of increased risk for chromosomal abnormalities (see Table 1). In our study, the proportion of fetuses with normal chromosomal profiles in the first trimester was 48.1%, a finding consistent with previously reported rates by Johnson et al. [6], Shulman et al. [29], and Rieteke et al. [35].

Of the thirteen pregnancies in our study that continued with a confirmed normal chromosomal complement, ten resulted in term deliveries without phenotypic signs/manifestations of cystic hygroma or some other structural abnormalities. Our findings support the view that fetuses with normal karyotypes that present with simple hygromas in the first trimester (particularly if not complicated by hydrops) frequently experience spontaneous resolution by weeks 14 to 16 of gestation and are likely to be normal at birth from the phenotypical perspective. Until more definitive data become available regarding potential structural anomalies in such instances, we would endorse detailed follow-up imaging, which includes fetal echocardiography, during the second trimester to ensure comprehensive evaluation.

Chromosomal microarray analysis (CMA) is a relatively recent genomic technique used to detect DNA copy number variations (CNVs) across the entire genome [35,36]. It is currently considered the first-line genetic test for children presenting with developmental delays, intellectual disabilities, congenital anomalies, or autism spectrum disorders [36,37]. In prenatal diagnostics, comparative genomic hybridization (CGH), a specific form of CMA, has been shown to reveal other clinically relevant chromosomal abnormalities in about 6% of fetuses with structural anomalies detected by ultrasonography, but with a conventional, normal karyotype [23,24,25]. Therefore, CGH is particularly advocated when one (or more) major structural abnormalities are discovered prenatally.

In our study, copy number variants were analyzed in 15 fetuses (7.3%) presenting with cystic hygroma in combination with other structural malformations. Pathogenic CNVs were identified in 5 of these 15 cases (33.3%) (Table 1). The pathological findings included 22q11.2 duplication, 22q11.2 microdeletion syndrome, a 10q26–12q26.3/12q21–q22 deletion, a 5q21.1–q34 deletion, and a terminal X chromosome deletion: 46,XX,del(X)(p11.3p22.3). Noonan syndrome, which is also associated with cystic hygromas appearing in the first trimester, was diagnosed in five fetuses in our cohort. In such cases, diagnostic work-up typically entails standard karyotyping, chromosomal microarray, and a targeted Noonan syndrome gene panel.

Recently, Norton et al. [24] conducted a secondary analysis of a study examining exome sequencing in instances of non-immune hydrops fetalis (NIHF). The analysis involved phenotype-driven exome sequencing in 127 affected fetuses. Pathogenic or potentially pathogenic variants were identified in 37 out of 127 cases (29%), spanning 29 genes in total. Additionally, variants of uncertain significance (VUS) that were strongly suspected to be associated with the clinical phenotype were found in a further 12 cases (9%). The authors noted that, had the NIHF cohort undergone sequencing using a limited gene panel, we could expect only between 19 and 24 pathogenic variants to be discovered. In contrast, the broader approach of exome sequencing achieved a diagnostic yield of 29%, compared to an estimated 18% with the largest available NIHF gene panel.

Conversely, Mellis et al. [38] assessed the utility of exome sequencing in the prenatal period to assess increased nuchal translucency (NT) in 213 pregnancies where fetuses exhibited NT measurements over 3.5 mm between 11 and 14 weeks of gestation. Diagnostic variants were found in 22.2% of fetuses (12 out of 54) where increased non-isolated NT was observed, and in 32.4% of fetuses (12 out of 37) where increased isolated NT was detected, together with other anomalies found later in pregnancy. However, only 2 of 11 (18.2%) fetuses with truly isolated increased NT in the first trimester, but without subsequent anomalies, had diagnostic findings. These authors concluded that while the diagnostic success rate of prenatal exome sequencing is limited in cases involving isolated increased NT, it increases substantially when additional fetal malformations are present later in gestation. A detailed comparison of our results with the available literature is presented in Table 2.

An often-overlooked aspect of prenatal diagnosis of cystic hygroma is the psychological impact on expectant parents following early detection of fetal anomalies. Studies have demonstrated that such diagnoses can lead to symptoms of depression, anxiety, and post-traumatic stress, contributing to considerable psychological distress. For instance, Fonseca et al. [39] found that parents receiving a prenatal diagnosis of a congenital anomaly exhibited increased levels of psychological distress compared to those whose infants were healthy—with mothers reporting more adjustment difficulties than fathers. Similarly, Bekkhus et al. [40] observed that expectant fathers experienced elevated psychological distress following the detection of fetal anomalies, underscoring the need for psychological support for both parents during this challenging period. Consequently, this should be integrated into prenatal care protocols to address the emotional needs of parents facing such diagnoses.

**Table 2:** An overview of chromosomal abnormalities identified in fetuses with first-trimester cystic hygroma, with comparison to reported frequencies in the literature.

Study	Number of Cases	Mean Gestational Age (Weeks)	Total
Reus et al., 1987 [41]	1	12	1/1 (100.0%)
Pons et al., 1989 [42]	4	11	4/4 (100.0%)
Bronshtein et al., 1989 [34]	70	12.4	10/70 (14.3%)
Cullen et al., 1990 [27]	30	10.6	15/30 (50.0%)
Podobnik et al., 1991 [11]	2	12.4	2/2 (100.0%)
Shulman et al., 1992 [29]	32	11.2	15/32 (46.9%)
Rietke et al., 1992 [35]	22	13.1	7/22 (31.8%)
Johnson et al., 1993 [6]	68	11.9	41/68 (60.3%)
Malone et al., 2005 [20]	134	12.5	67/134 (50.0%)
Podobnik et al., 2008 [22]	35	11.8	17/35 (48.6%)
Scholl et al., 2016 [25]	212	12.3	128/212 (60.4%)
Sparks et al., 2020 [23]	127	13	37/127 (30% with pathological CMA) (29.1%)
Malone et al., 2021 [7]	410	12.8	230/410 (56.1%)
Current study	405	11.5	210/405 (51.9%)
Total	1552	11.8	784/1552 (50.5%)

Importantly, in our study a subset of fetuses with isolated, spontaneously resolving hygromas and normal genetic findings had favorable outcomes, highlighting the prognostic value of combining detailed imaging with genetic testing. These findings further emphasize the importance of tailored counseling for expectant parents, balancing reassurance in favorable cases with vigilance for potential underlying conditions. Still, the potential for underlying sub-microscopic anomalies underscores the need for expanding access to advanced molecular diagnostics, particularly in cases where standard karyotyping is inconclusive.

This study has several strengths. It is based on a large number of pregnancies evaluated over a thirty-year period, which goes towards improved reliability and generalizability of the results/conclusions. The use of high-resolution 3D/4D transvaginal ultrasonography allowed for accurate and early detection of fetal cystic hygroma, while the integration of comprehensive genetic testing provided a thorough genetic assessment. The study also offers valuable insight by correlating ultrasonographic characteristics (such as the presence or absence of septations) with fetal chromosomal outcomes and postnatal findings. Nevertheless, there are also certain shortcomings. A retrospective design of the study imposes inherent limitations, including potential variability in documentation and diagnostic protocols over the extended study period. We also had limited ability to detect pathogenic variants beyond those identifiable by standard microarray or targeted panels, and the absence of systematic postnatal genetic follow-up in euploid cases hampers the detection of subtle or late-onset genetic syndromes. Postnatal follow-up was limited in terminated pregnancies and in cases without overt anomalies at birth, making long-term outcome data incomplete. Also, stratification of results by imaging modality or time period was not feasible due to gradual and overlapping changes in technology, practice and referral patterns; hence, future prospective, multicentre studies should be designed to capture such information in a standardized manner to enable robust temporal and modality-based comparisons. Nonetheless, this is the first and largest study in Croatia with a comprehensive prenatal and genetic profile of fetal cystic hygroma over such an extended period, offering valuable insights into its diagnostic, prognostic, and clinical management implications.

## 5. Conclusions

This study confirms that fetal cystic hygroma, particularly when identified in the first or early second trimester of pregnancy, is a significant prenatal marker linked to chromosomal abnormalities and undesired perinatal outcomes. Our findings demonstrate a strong correlation between early ultrasonographic detection (whether septated or non-septated) and the presence of chromosomal aneuploidies, structural malformations, as well as genetic syndromes such as Noonan syndrome. The detection of cystic hygroma, even without any additional anomalies, warrants a thorough diagnostic work-up including cytogenetic analysis, chromosomal microarray and, where appropriate, targeted gene panels.

Given the variability in outcomes, the management of pregnancies complicated by cystic hygroma should be multidisciplinary, involving fetal medicine specialists, genetic counselors and neonatologists. Health systems should ensure equitable access to advanced genetic testing and fetal imaging to improve diagnostic accuracy and parental decision-making. Investing in clinician education and standardized sonographic protocols may also contribute to earlier and more reliable detection across different healthcare settings—and ultimately contribute to achieving better prognoses.

## Figures and Tables

**Table 1:** The breakdown of chromosomal and structural abnormalities linked to first-trimester diagnoses of fetal cystic hygroma, presented in both absolute numbers and corresponding percentages (*N* = 210).

Category	Count (*N*)	Percentage (%)
Total chromosomal abnormalities	210	100 (51.9 as a fraction of all pregnancies)
Trisomy 21	80	38.1
Trisomy 18	47	22.4
Trisomy 13	33	16.6
Turner syndrome (45,X)	30	14.2
Turner mosaic	3	1.4
Copy number variants (total)	5	2.3
10q26.12q26.3 deletion	1	0.4
5q21.1q34 deletion	1	0.4
22q11.2 deletion	1	0.4
22q11.2 duplication	1	0.4
46,XX, del(2q13)	1	0.4
Other syndromes	7	3.3
Noonan syndrome	5	2.4
Roberts syndrome	1	0.4
Cornelia de Lange syndrome	1	0.4
Structural anomalies (in abnormal karyotypes)	15	7.1
Hydrops, effusion, ascites	6	2.9
Hydrocephalus	2	1.0
Arthrogryposis	1	0.4
Agenesis of corpus callosum	1	0.4
Diaphragmatic hernia	1	0.4
Meromelia	1	0.4
Tetralogy of Fallot	2	1.0
Bilateral hydronephrosis	1	0.4

## Data Availability

Original dataset available upon reasonable request from the authors.
